# The search for novel analgesics: re-examining spinal cord circuits with new tools

**DOI:** 10.3389/fphar.2014.00022

**Published:** 2014-02-25

**Authors:** Kelly M. Smith, Jessica F. Madden, Robert J. Callister, David I. Hughes, Brett A. Graham

**Affiliations:** ^1^School of Biomedical Sciences and Pharmacy, Faculty of Health and Medicine, Hunter Medical Research Institute, University of NewcastleNewcastle, NSW, Australia; ^2^Institute of Neuroscience and Psychology, University of GlasgowGlasgow, UK

**Keywords:** dorsal horn, interneuron, optogenetics, genetic profiling

## Abstract

In this perspective, we propose the absence of detailed information regarding spinal cord circuits that process sensory information remains a major barrier to advancing analgesia. We highlight recent advances showing that functionally discrete populations of neurons in the spinal cord dorsal horn (DH) play distinct roles in processing sensory information. We then discuss new molecular, electrophysiological, and optogenetic techniques that can be employed to understand how DH circuits process tactile and nociceptive information. We believe this information can drive the development of entirely new classes of pharmacotherapies that target key elements in spinal circuits to selectively modify sensory function and blunt pain.

## INTRODUCTION

Pain is an important percept within our somatosensory system. It provides an alert to actual or potential tissue damage and ensures injured tissue is protected during healing (Basbaum et al., 2009). Despite its important biological function, the unpleasant nature of pain and its tendency to often outlast the initial stimulus has driven a search for strategies to relieve it for many millennia. For example, Sumerian clay tablets (~3400 BC) refer to opium cultivation for pain relief, and to this day opium derivatives remain the gold standard for treating moderate and severe pain (Rosenblum et al., 2008). The fact that an age-old analgesic remains at the frontline of pain treatment emphasizes the slow progress in analgesic research. Together with the problematic side effects of opiates, their addiction and abuse potential, and the “opiate resistance” of many chronic pain conditions (including neuropathic pain) these factors reinforce the urgent need to identify and develop new analgesics (de Leon-Casasola, 2013).

The neuronal pathways that transmit nociceptive signals to the brain contain multiple sites that present opportunities to pharmacologically alter signaling and ultimately influence or block the pain experience. This complex task of sensing, encoding and perceiving stimuli that could generate pain begins with the collection of information in peripheral sensory receptors, termed nociceptors. Nociceptors are located throughout our body in skin, muscle, joints and viscera, forming diverse populations that can encode either exclusively or combinations of high intensity thermal, mechanical and chemical stimuli. The mechanisms that lead to nociceptor activation and sensitisation obviously provide potential analgesic targets [for review see (Stein et al., 2009; Richards and McMahon, 2013)]. However, targeting peripheral components of the nociceptive system has little value in pain conditions where aberrant signaling, in the form of hyperactive neuron populations and circuits, is firmly established in the central nervous system (CNS).

Here we focus on the spinal cord dorsal horn (DH), the first site where nociceptive information enters the CNS, is processed, and subsequently relayed through successively higher levels of the neuroaxis to form a sensory percept. The necessity for nociceptive information to pass through the DH and ascend to higher brain structures before pain is experienced makes it an attractive site for pharmacological targeting. Indeed, this has been well accepted since publication of the gate control theory of pain by Melzack and Wall (1965). Furthermore, we now know that both peripheral and central insults can disrupt normal information flow through the neuroaxis by initiating reorganization of circuits in the spinal cord DH, brainstem, thalamus, limbic, or cortical regions and produce altered sensory perception in conditions such as neuropathic pain or itch (Graham and Callister, 2012). Given this longstanding focus it is not unreasonable to ask, “Why has progress in spinal-based analgesics been so slow”?

We believe an answer to this question may lie in our overly simplified view of the DH. The general view is that this spinal region acts as a single processing unit, even though we know it receives diverse signals from thermal, nociceptive, pruritic (itch), and tactile peripheral receptors. Thus, neurons in the DH must simultaneously fulfil several roles, which are critical for normal sensory experience: i.e., the integration of different types of signals and segregation of others into specific ascending pathways (Todd, 2010). A classic example is the segregation of nociceptive and tactile information in the DH. This ensures peripheral stimuli such a sharp pin-prick or light touch result in very different and contextually relevant sensory experiences. One of the critical substrates for these processing tasks is the diversity of neuron types, which form DH circuits and have very specific properties. Surprisingly, our knowledge of the discrete neuronal types within DH circuits and their precise role in sensory processing is limited.

These gaps in our knowledge exist because of historical limitations in experimental approaches. Until recently spinal cord researchers have generally been forced to collect data from multiple (unidentified) neuronal classes and then “pool” these results to provide an “averaged view” of sensory processing and function (or dysfunction). This approach clearly overlooks cell-type diversity in the DH as originally observed by Ramon y Cajal (1899) more than a century ago. Since Cajal’s work there has been general agreement that if we are to understand nervous system function, we must first understand how neuron types are assembled into processing circuits. Armed with this mechanistic understanding, we may then identify putative drug targets on specific neuron types. We believe this approach offers the promise of targeted analgesics that selectively act on nociceptive circuits.

At the same time, it is important to acknowledge that the difficulty in defining functionally discrete neuron populations within the DH is not due to a lack of effort. In fact, an extensive literature shows that multiple neuron classes do exist as based on any single parameter (e.g., electrophysiology, neurochemistry, morphology). For example, inhibitory interneurons in the superficial DH can be differentiated into four populations based on the non-overlapping expression of neurochemical markers (neuropeptide Y, galanin, parvalbumin, and nitric oxide synthase; Polgár et al., 2013). The same approach reveals up to six populations of excitatory interneurons (calretinin, calbindin, neurotensin, somatostatin, substance P, and neurokinin B; Todd, 2010), though some overlap exists within expression patterns. In contrast, electrophysiological classification distinguishes four to seven types of neurons based on action potential discharge patterns during depolarizing current injection (Grudt and Perl, 2002; Ruscheweyh and Sandkuhler, 2002; Graham, 2004). Finally, using anatomical criteria, four distinct morphologies are commonly differentiated (Grudt and Perl, 2002; Yasaka et al., 2010). The challenge has been to merge this information into a model that defines neuron populations with homogenous properties based on multiple criteria. Only with this information can we begin to understand how DH circuits process both nociceptive and non-nociceptive information and develop tools that allow us to manipulate this region and provide pain relief.

## SPINAL SUBPOPULATIONS MATTER FOR PAIN PROCESSING IN THE DH

Some evidence is now accumulating to support a key role for functionally and neurochemically distinct neuronal populations in spinal sensory processing. For example, recent work has examined the role of excitatory DH interneurons in pain and itch. In these experiments knockout of the testicular orphan nuclear receptor 4 (TR4) in the CNS resulted in a substantial loss (~70%) of excitatory interneurons (Wang et al., 2013). Behavioral analyses then identified an almost complete loss of supraspinally mediated pain and itch responses, elevated mechanical withdrawal thresholds, and nerve injury-induced mechanical hypersensitivity. In contrast, noxious heat evoked reflexes that originate in the spinal cord, nerve injury-induced heat hypersensitivity, and tissue injury-induced heat and mechanical hypersensitivity were unaltered. The authors concluded, “that different subsets of dorsal excitatory interneurons contribute to tissue and nerve injury-induced heat and mechanical pain” (Wang et al., 2013). This study complements a larger body of work, which began with the gate control theory of pain, implicating populations of inhibitory (GABA and glycine containing) DH interneurons in nociceptive processing (Zeilhofer et al., 2011). This work has firmly established that inhibitory dysfunction allows “linking” of tactile and nociceptive circuits to produce allodynia and hyperalgesia. The search for specific neuronal subpopulations directly involved in this process has unfortunately progressed slowly.

Despite the above challenges, work on inhibitory interneurons has succeeded in identifying a functionally distinct neuronal subpopulation in the DH (Ross et al., 2010). This work, which is relevant to itch rather than nociception, assessed the role of a particular transcription factor – Bhlhb5. Mice lacking Bhlhb5 exhibited an itch phenotype and lacked a subset of inhibitory interneurons in the DH. Importantly, the remaining neuronal populations, afferent input, and responses to other sensory modalities were not altered. This confirmed a specific role for the lost inhibitory population in the processing of itch-related stimuli. Together with the data on excitatory populations, this work provides support for the hypothesis that additional unidentified interneuron subpopulations exist in the DH.

## TARGETING SPINAL SUBPOPULATIONS

Over the last decade, transgenic techniques have been developed which allow marker proteins such as green fluorescent protein (GFP) to be expressed in specific neurons, thereby enabling us to visualize and target specific neuronal subtypes in the CNS. In simple terms, this is achieved by genetic techniques that couple GFP expression to a promoter protein that only exists in neurons of interest. As long as we have a “genetic signature” for a given neuron type, this approach allows us to address DH interneuron diversity. To date, such studies have been restricted to inhibitory interneurons whereby GFP expression has been linked to proteins involved in neurotransmitter synthesis and membrane transport (Heinke, 2004; Zeilhofer et al., 2004). This work has, however, still reported significant variability in the properties of targeted populations in GFP-positive neurons. Nevertheless, specifically studying GABAergic interneurons labeled by GFP has produced important findings. For example, a small subpopulation of GFP-positive neurons has been identified that receive low-threshold (tactile) input from primary afferents (Daniele and MacDermott, 2009). The ability of these tactile inputs to activate GABAergic interneurons has long been acknowledged as a basic requirement for inhibitory circuits to “segregate” nociceptive and tactile information. While such transgenic approaches provide a powerful tool to study subpopulations of neurons, it seems identifying specific target proteins to drive GFP expression in these populations (i.e., the neuron’s genetic “signature”) is challenging (Graham et al., 2007). For example if the aim is to identify discrete classes of neurons with uniform properties and clear roles in sensory processing, labeling neurons based upon the primary neurotransmitter they employ appears too crude an approach.

**FIGURE 1:**
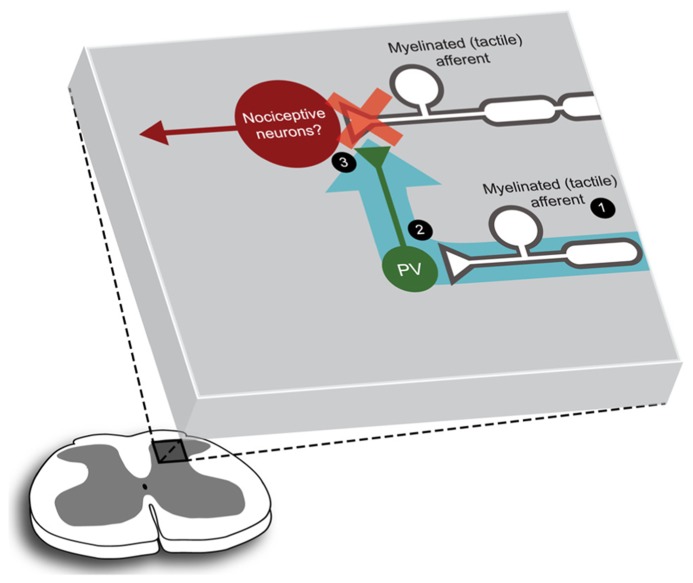
**Parvalbumin-positive DH neurons are configured to mediate presynaptic control of myelinated afferent fiber that contribute to DH circuits.** Figure summarizes a characterization of PV-positive neurons using a GFP expressing transgenic mouse line (Hughes et al., 2012). PV-positive DH neurons and their axonal arbors (2, green) are concentrated in lamina IIi/III. These cells receive input from myelinated (tactile) primary afferents (1), and their axons appear to target the central terminals of myelinated primary afferents preferentially, forming inhibitory axoaxonic synaptic inputs (3). Thus, when this microcircuit is activated (blue arrow) myelinated afferent input is inhibited, or gated (red cross). The downstream target of this PV-gated input remains to be identified, however, the location of these connections suggests that neurons in nociceptive circuits may be included.

With these challenges in mind, we have recently characterized a small but significant subpopulation of inhibitory interneurons that express the calcium-binding protein parvalbumin (PV; Hughes et al., 2012). This work places PV-positive interneurons in a putative circuit for mediating feed-forward inhibition in the DH (**Figure 1**). Using GFP to target PV-expressing neurons we showed that the functional and morphological properties of PV-positive interneurons were remarkably homogeneous. PV-positive interneurons exhibited excitable, high frequency AP discharge responses and typically had islet like morphologies. Furthermore, a significant proportion (~80%) of axons arising from PV-positive DH neurons made selective axoaxonic-synapses onto the central terminals of myelinated afferents. We also showed that PV-positive interneurons received input from the same myelinated afferent population. This connectivity is ideally suited to maintain functional segregation of sensory modalities. Thus, under normal conditions when tactile related information arrives in the DH, PV-neurons are excited and subsequently inhibit tactile inputs. This action prevents further excitation that would otherwise recruit nociceptive circuits (**Figure 1**). By extension, decreased PV-neuron excitability would remove this gating of tactile information, link tactile and nociceptive signaling and produce tactile allodynia. An important caveat to this work is that despite the success of our PV targeting approach, some heterogeneity remained in our sample. Specifically, 20% of the axons arising from PV-positive neurons targeted unidentified structures and some variability remained in our electrophysiological and morphological data. In summary, our findings in the PV-GFP mouse, along with earlier GFP studies, suggest additional analyses are required to uncover more discrete neuronal subpopulations and determine their functional role in spinal sensory processing.

## NEW TOOLS TO DEFINE FUNCTIONALLY DISCRETE SUBPOPULATIONS

Fortunately, a number of techniques are becoming available which could expand our analysis of DH neuron subpopulations. For example, several groups have used molecular screening techniques to dissect neuronal heterogeneity. This has only recently been applied to the DH (Wildner et al., 2013). Here two transgenic mice, which lacked key transcriptional regulators that normally define inhibitory DH interneuron lineages, were subjected to molecular screening. Genome-wide expression comparisons identified four genes (pDyn, Kcnip2, Rorb, and Tfap2b) with largely non-overlapping expression patterns that were significantly down regulated in the DH of animals lacking inhibitory interneuron populations (Wildner et al., 2013). The group is now testing how subpopulations that selectively express these four genes contribute to sensory and nociceptive processing in the DH. A variation of this strategy has also been applied to a number of CNS regions whereby gene expression profiling is undertaken in individual, or small numbers of neurons identified via GFP expression. This approach builds on GFP targeting studies by using single-cell quantitative PCR (qPCR) to compare gene expression profiles in subsets of identified neurons. This establishes smaller groupings of neurons within a GFP labeled subpopulation that can be considered distinct according to molecular criteria. Such information can then be used to predict the function of different subpopulations as well as providing novel electrophysiological and anatomical signatures to identify these populations in subsequent studies. The technique also identifies a series of “marker” genes and proteins that can be used to subdivide, label, monitor, and manipulate subpopulations of interest. This procedure has proved valuable in other sensory processing nodes such as the medial vestibular nucleus (MVN; Sugino et al., 2005; Kodama et al., 2012). Gene expression profiling in the MVN identified six distinct neuronal subpopulations. Subsequent combination of this information with electrophysiological and anatomical data allowed several populations to be assigned specific functions in vestibulo-ocular and vestibulo-cerebellar circuits. The DH represents an ideal candidate for this type of analysis as the functional significance of its neuron heterogeneity is yet to be understood and several DH GFP labeled populations are now available. We believe that such studies could identify novel protein targets with a high degree of specificity in terms of the number and identity of DH neurons that could be manipulated and blunt pain.

Once molecular screening has been used to identify specific protein targets in discrete neuronal subpopulations, the next critical step is to determine their relevance and role in nociceptive signal processing (versus other modalities) by establishing their connectivity. Until recently it has been virtually impossible to obtain such information for complex neural circuits. The best data for the DH has comes from electrophysiological recordings from pairs of “synaptically connected” neurons in spinal cord slices. These experiments are difficult and typically result in small sample sizes, especially in the DH where connectivity rates are low in slices – [10–15% (Lu and Perl, 2003, 2005)]. More recently a series of elegant publications by the Strassman group have employed laser-scanning microstimulation to investigate connectivity in the DH describing fundamental principles for DH synaptic connectivity patterns (Kato et al., 2009; Kosugi et al., 2013). Notwithstanding the substantial advances this approach has delivered, laser-scanning microstimulation only allows for an identified neuron’s inputs to be studied without providing information on the type or types of neurons where these inputs originate. Fortunately, connectivity mapping has recently been revolutionized with the introduction of optogenetic techniques. This approach allows neurons with the “same genetic signature” to be activated by light. Specifically, light-sensitive proteins (channel rhodopsins from algae) are incorporated into a given neuron type. When stimulated by light of an appropriate wavelength, the channel rhodopsins either promote or inhibit neuron activity (Fenno et al., 2011). Optogenetics allows selective activation of neurons within given circuits and vastly increases the speed and accuracy of connectivity studies.

So far optogenetics has not yet been used to study neuronal connectivity within DH circuits. Channel rhodopsins have, however, been expressed in a subpopulation of polymodal unmyelinated (Mrgprd-positive) sensory afferents (Wang and Zylka, 2009). DH neuron responses were recorded in spinal cord slices during selective “light activation” of Mrgprd-positive unmyelinated afferents. This is significant as there are several classes of polymodal afferents and it is not possible to selectively stimulate them electrically. The results of this work showed that Mrgprd-positive afferents provide relatively broad and non-selective input to multiple DH populations, reinforcing a major premise of this perspective – i.e., that functionally discrete DH neuronal populations are critical for appropriate encoding of sensory information. ChR2 has also been expressed in Nav1.8 positive afferent neurons, a manipulation that provides optogenetic control of almost all nociceptors (Daou et al., 2013). In contrast to the Mrgprd experiments, this work was undertaken *in vivo*. Light stimulation of the hindpaw resulted in pain behaviors (i.e., foot withdrawal). These data provide the first proof of concept that optogenetics can be employed under both *in vivo* and *in vitro* conditions and highlights the potential for these approaches to be used in development and testing of novel pain therapeutics.

## SUMMARY AND CONCLUSION

**FIGURE 2:**
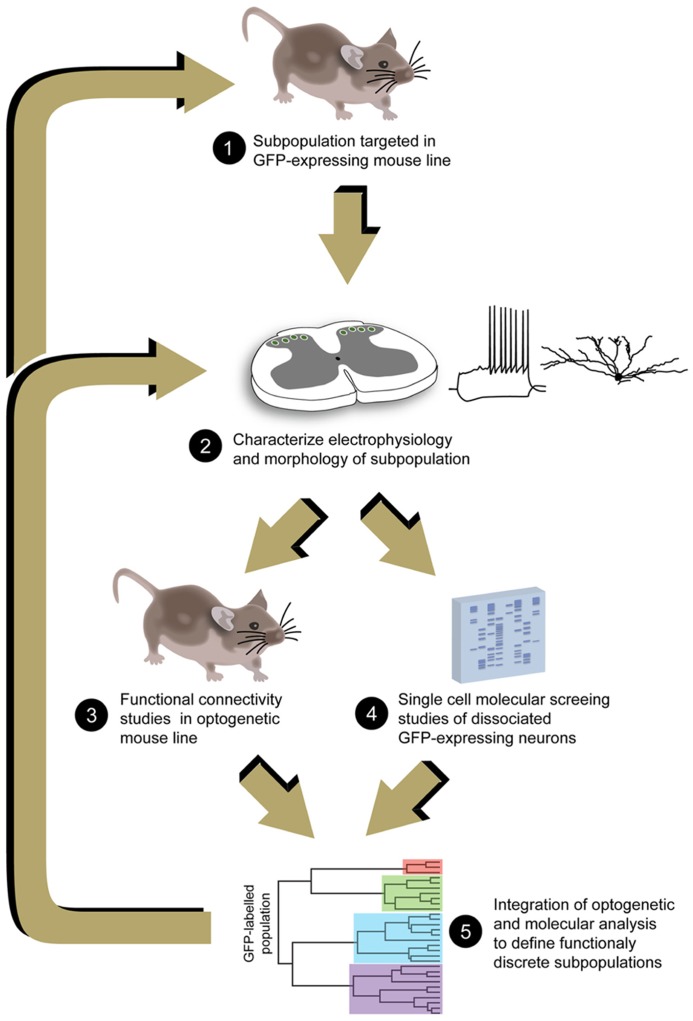
**Proposed analysis pathway to resolve DH neuron heterogeneity.** Analyses begin with identification of GFP expressing transgenic mouse lines that label DH neuron populations (1). These neurons are first characterized using electrophysiological and anatomical approaches, determining the degree of homogeneity/heterogeneity that exists in the GFP-expressing population (2). Additional transgenic mice can then be employed that express optogenetic probes to selectively activate the subpopulations of interest and study their connectivity in DH circuits (3). GFP-expressing populations can also be subjected to gene expression profiling to further dissect any remaining heterogeneity and identify novel proteins that are selectively expressed in these subpopulations (4). This information can then be fed back into the analysis strategy, further refining the selection of probes to target smaller DH subpopulations, and identifying unique function and neuroanatomical features that can then be used to identify these subpopulations.

The challenge of unraveling cellular heterogeneity in the DH remains a major barrier to understanding how this region encodes our sensory world. The value of this sort of analysis in the CNS has been emphasized in a recent review that reinforced Cajal’s original premise: “A complete understanding of nervous system function cannot be achieved without the identification of its component cell types” (Fishell and Heintz, 2013). Our failure to meet this challenge has been largely due to technical limitations, however, a range of leading-edge techniques including molecular phenotyping of individual neurons and optogenetic activation of specific neuronal populations now allows us to proceed (**Figure 2**). In addition, other techniques such as chemogenetic approaches, also referred to as designer receptors exclusively activated by designer drug (or DREADD), are likely to form part of the ever-increasing armory available to manipulate and study the functional role of neurons in the DH (see Rogan and Roth, 2011; Wess et al., 2013). Importantly, the application of these technologies in pain research will require continued advances in molecular probe development, techniques and equipment to delivery light stimulation to the spinal cord. The fundamental information provided by these new techniques will inform future attempts to treat a variety of painful conditions in which sensory processing and perception are disrupted in the DH. For example our dataset on PV-positive DH neurons suggest they play a key role in separating tactile and nociceptive information in the spinal cord. Thus, targetted activation of this functionally discrete DH population may reduce tactile allodynia, a feature of many chronic pain syndromes where tactile sensory input excites nociceptive circuits and produces pain. The information from molecular screening and optogenetic analysis of these DH neurons ultimately aims to identify pharmacological agents that are capable of restoring normal sensory function by abolishing tactile allodynia. Likewise, as additional functionally discrete DH subpopulations are characterized this same strategy could lead to new classes of analgesic drugs with very specific actions.

## Conflict of Interest Statement

The authors declare that the research was conducted in the absence of any commercial or financial relationships that could be construed as a potential conflict of interest.

## References

[B1] Basbaum A. I. Bautista D. M. Scherrer G. Julius D. (2009). Cellular and molecular mechanisms of pain. *Cell* 139 267–284 10.1016/j.cell.2009.09.028 19837031 PMC2852643

[B3] Daniele C. A. MacDermott A. B. (2009). Low-threshold primary afferent drive onto GABAergic interneurons in the superficial dorsal horn of the mouse. *J. Neurosci.* 29 686–695 10.1523/JNEUROSCI.5120-08.2009 19158295 PMC2826179

[B4] Daou I. Tuttle A. H. Longo G. Wieskopf J. S. Bonin R. P. Ase A. R. (2013). Remote optogenetic activation and sensitization of pain pathways in freely moving mice. *J. Neurosci.* 33 18631–18640 10.1523/JNEUROSCI.2424-13.2013 24259584 PMC6618811

[B5] de Leon-Casasola O. A. (2013). Opioids for chronic pain: new evidence, new strategies, safe prescribing. *Am. J. Med.* 126 S3–S11 10.1016/j.amjmed.2012.11.011 23414718

[B6] Fenno L. Yizhar O. Deisseroth K. (2011). The development and application of optogenetics. *Annu. Rev. Neurosci.* 34 389–412 10.1146/annurev-neuro-061010-113817 21692661 PMC6699620

[B7] Fishell G. Heintz N. (2013). The neuron identity problem: form meets function. *Neuron* 80 602–612 10.1016/j.neuron.2013.10.035 24183013

[B8] Graham B. A. (2004). In vivo responses of mouse superficial dorsal horn neurones to both current injection and peripheral cutaneous stimulation. *J. Physiol*. 561 749–763 10.1113/jphysiol.2004.072645 15604230 PMC1665382

[B9] Graham B. Callister R. (2012). “Pain,” in *The Mouse Nervous System* eds Watson C. Paxinos G. Puelles L. (London: Elsevier) 589–606

[B10] Graham B. A. Brichta A. M. Callister R. J. (2007). Moving fom an averaged to specific view of spinal cord pain processing circuits. *J. Neurophysiol.* 98 1057–1063 10.1152/jn.00581.2007 17567772

[B11] Grudt T. J. Perl E. R. (2002). Correlations between neuronal morphology and electrophysiological features in the rodent superficial dorsal horn. *J. Physiol.* 540 189–207 10.1113/jphysiol.2001.012890 11927679 PMC2290200

[B12] Heinke B. (2004). Physiological, neurochemical and morphological properties of a subgroup of GABAergic spinal lamina II neurones identified by expression of green fluorescent protein in mice. *J. Physiol.* 560 249–266 10.1113/jphysiol.2004.070540 15284347 PMC1665197

[B13] Hughes D. I. Sikander S. Kinnon C. M. Boyle K. A. Watanabe M. Callister R. J. (2012). Morphological, neurochemical and electrophysiological features of parvalbumin-expressing cells: a likely source of axo-axonic inputs in the mouse spinal dorsal horn. *J. Physiol.* 590 3927–3951 10.1113/jphysiol.2012.235655 22674718 PMC3476641

[B14] Kato G. Kawasaki Y. Koga K. Uta D. Kosugi M. Yasaka T. (2009). Organization of intralaminar and translaminar neuronal connectivity in the superficial spinal dorsal horn. *J. Neurosci.* 29 5088–5099 10.1523/JNEUROSCI.6175-08.2009 19386904 PMC2777732

[B15] Kodama T. Guerrero S. Shin M. Moghadam S. Faulstich M. du Lac S. (2012). Neuronal classification and marker gene identification via single-cell expression profiling of brainstem vestibular neurons subserving cerebellar learning. *J. Neurosci.* 32 7819–7831 10.1523/JNEUROSCI.0543-12.2012 22674258 PMC3410725

[B16] Kosugi M. Kato G. Lukashov S. Pendse G. Puskar Z. Kozsurek M. (2013). Subpopulation-specific patterns of intrinsic connectivity in mouse superficial dorsal horn as revealed by laser scanning photostimulation. *J. Physiol.* 591 1935–1949 10.1113/jphysiol.2012.244210 23297304 PMC3624861

[B17] Lu Y. Perl E. R. (2003). A specific inhibitory pathway between substantia gelatinosa neurons receiving direct C-fiber input. *J. Neurosci.* 23 8752–8758 14507975 10.1523/JNEUROSCI.23-25-08752.2003 PMC6740424

[B18] Lu Y. Perl E. R. (2005). Modular organization of excitatory circuits between neurons of the spinal superficial dorsal horn (Laminae I and II). *J. Neurosci.* 25 3900–3907 10.1523/JNEUROSCI.0102-05.2005 15829642 PMC6724918

[B19] Melzack R. Wall P. D. (1965). Pain mechanisms: a new theory. *Science* 150 971–979 10.1126/science.150.3699.971 5320816

[B20] Polgár E. Sardella T. C. P. Tiong S. Y. X. Locke S. Watanabe M. Todd A. J. (2013). Functional differences between neurochemically defined populations of inhibitory interneurons in the rat spinal dorsal horn. *Pain* 154 2606–2615 10.1016/j.pain.2013.05.001 23707280 PMC3858808

[B2] Ramon y Cajal S. (1899). *Histology of the Nervous System.* Swanson N. Swanson L. W. (trans.) (Wien, New York: Springer)

[B21] Richards N. McMahon S. B. (2013). Targeting novel peripheral mediators for the treatment of chronic pain. *Br. J. Anaesth.* 111 46–51 10.1093/bja/aet216 23794644

[B22] Rogan S. C. Roth B. L. (2011). Remote control of neuronal signaling. *Pharmacol. Rev.* 63 291–315 10.1124/pr.110.003020 21415127 PMC3082452

[B23] Rosenblum A. Marsch L. A. Joseph H. Portenoy R. K. (2008). Opioids and the treatment of chronic pain: controversies, current status, and future directions. *Exp. Clin. Psychopharmacol.* 16 405–416 10.1037/a0013628 18837637 PMC2711509

[B24] Ross S. E. Mardinly A. R. McCord A. E. Zurawski J. Cohen S. Jung C. (2010). Loss of inhibitory interneurons in the dorsal spinal cord and elevated itch in bhlhb5 mutant mice. *Neuron* 65 886–898 10.1016/j.neuron.2010.02.025 20346763 PMC2856621

[B25] Ruscheweyh R. Sandkuhler J. (2002). Lamina-specific membrane and discharge properties of rat spinal dorsal horn neurones in vitro. *J. Physiol.* 541 231–244 10.1113/jphysiol.2002.017756 12015432 PMC2290304

[B26] Stein C. Clark J. D. Oh U. Vasko M. R. Wilcox G. L. Overland A. C. (2009). Peripheral mechanisms of pain and analgesia. *Brain Res. Rev.* 60 90–113 10.1016/j.brainresrev.2008.12.017 19150465 PMC2730351

[B27] Sugino K. Hempel C. M. Miller M. N. Hattox A. M. Shapiro P. Wu C. (2005). Molecular taxonomy of major neuronal classes in the adult mouse forebrain. *Nat. Neurosci.* 9 99–107 10.1038/nn1618 16369481

[B28] Todd A. J. (2010). Neuronal circuitry for pain processing in the dorsal horn. *Nat. Rev. Neurosci.* 11 823–836 10.1038/nrn2947 21068766 PMC3277941

[B29] Wang H. Zylka M. J. (2009). Mrgprd-expressing polymodal nociceptive neurons innervate most known classes of substantia gelatinosa neurons. *J. Neurosci.* 29 13202–13209 10.1523/JNEUROSCI.3248-09.2009 19846708 PMC2789299

[B30] Wang X. Zhang J. Eberhart D. Urban R. Meda K. Solorzano C. (2013). Excitatory superficial dorsal horn interneurons are functionally heterogeneous and required for the full behavioral expression of pain and itch. *Neuron* 78 312–324 10.1016/j.neuron.2013.03.001 23622066 PMC3700415

[B31] Wess J. Nakajima K. Jain S. (2013). Novel designer receptors to probe GPCR signaling and physiology. *Trends Pharmacol. Sci.* 34 385–392 10.1016/j.tips.2013.04.006 23769625 PMC3758874

[B32] Wildner H. Das Gupta R. Brohl D. Heppenstall P. A. Zeilhofer H. U. Birchmeier C. (2013). Genome-wide expression analysis of ptf1a- and ascl1-deficient mice reveals new markers for distinct dorsal horn interneuron populations contributing to nociceptive reflex plasticity. *J. Neurosci.* 33 7299–7307 10.1523/JNEUROSCI.0491-13.2013 23616538 PMC3684736

[B33] Yasaka T. Tiong S. Y. Todd A. J. (2010). Populations of inhibitory and excitatory interneurons in lamina II of the adult rat spinal dorsal horn revealed by a combined electrophysiological and anatomical approach. *Pain* 151 475–488 10.1016/j.pain.2010.08.008 20817353 PMC3170912

[B34] Zeilhofer H. U. Studler B. Arabadzisz D. Schweizer C. Ahmadi S. Layh B. (2004). Glycinergic neurons expressing enhanced green fluorescent protein in bacterial artificial chromosome transgenic mice. *J. Comp. Neurol.* 482 123–141 10.1002/cne.20349 15611994

[B35] Zeilhofer H. U. Wildner H. Yévenes G. E. (2011). Fast synaptic inhibition in spinal sensory processing and pain control. *Physiol. Rev.* 92 193–235 10.1152/physrev.00043.2010 22298656 PMC3590010

